# A Decade of Mighty Lipophagy: What We Know and What Facts We Need to Know?

**DOI:** 10.1155/2021/5539161

**Published:** 2021-11-05

**Authors:** Muhammad Babar Khawar, Muddasir Hassan Abbasi, Mussarat Rafiq, Naila Naz, Rabia Mehmood, Nadeem Sheikh

**Affiliations:** ^1^Molecular Medicine and Cancer Therapeutics Lab, Department of Zoology, Faculty of Sciences, University of Central Punjab, Lahore, Pakistan; ^2^Department of Zoology, University of Okara, Okara, Pakistan; ^3^Cell & Molecular Biology Lab, Department of Zoology, University of the Punjab, Q-A- Campus, Lahore 54590, Pakistan; ^4^Division of cell Matrix Biology and Regenerative Medicine, University of Manchester, Manchester, UK

## Abstract

Lipids are integral cellular components that act as substrates for energy provision, signaling molecules, and essential constituents of biological membranes along with a variety of other biological functions. Despite their significance, lipid accumulation may result in lipotoxicity, impair autophagy, and lysosomal function that may lead to certain diseases and metabolic syndromes like obesity and even cell death. Therefore, these lipids are continuously recycled and redistributed by the process of selective autophagy specifically termed as lipophagy. This selective form of autophagy employs lysosomes for the maintenance of cellular lipid homeostasis. In this review, we have reviewed the current literature about how lipid droplets (LDs) are recruited towards lysosomes, cross-talk between a variety of autophagy receptors present on LD surface and lysosomes, and lipid hydrolysis by lysosomal enzymes. In addition to it, we have tried to answer most of the possible questions related to lipophagy regulation at different levels. Moreover, in the last part of this review, we have discussed some of the pathological states due to the accumulation of these LDs and their possible treatments under the light of currently available findings.

## 1. Autophagy: A Brief Overview

**Figure 1:**
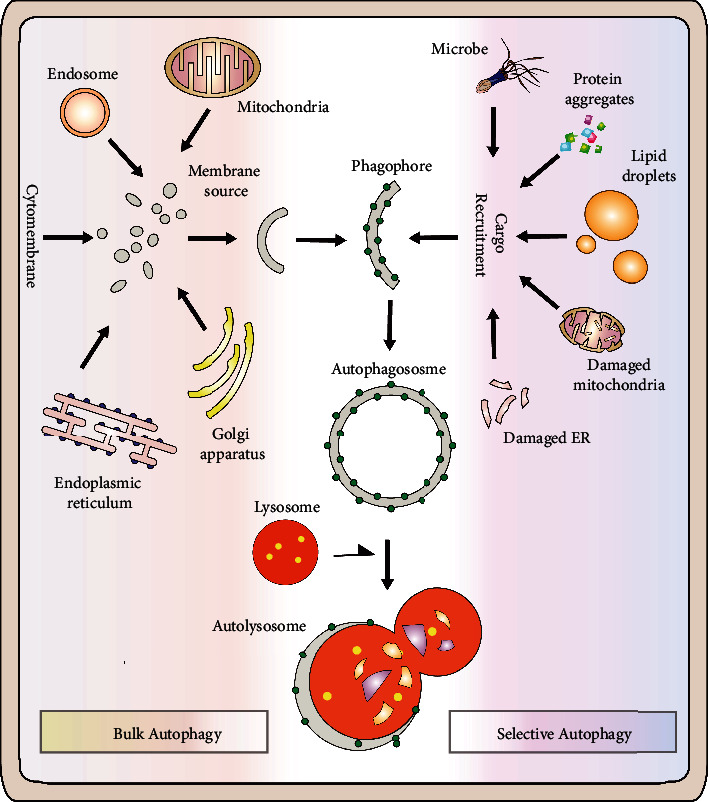
Bulk autophagy vs. selective autophagy: autophagy is a natural intracellular catabolic activity that helps cell in the removal of unwanted substances and recycling of cellular components while selective autophagy selectively target several specific cargos including various organelles, cellular substances, and protein aggregates.

Autophagy is a conserved intracellular degradative process conserved from very simple organisms (yeast) to highly complex animals (mammals). This process begins with the formation of a double-membrane cup-like structure named phagophore in response to stress circumstances (i.e., nutrient scarcity). In the subsequent step, autophagosome is formed as a result of an extension of the phagophore, sequestrate of cytoplasmic contents/organelles, and finally, the closure of both ends. Afterward, the resultant autophagosome is fused with the lysosome to form an autolysosome to finally break down the desired substance (Figure 1) [1–3]. Autophagy is not only important for supplying energy to starving cells; instead, it is very crucial for the smooth physiological integrity of the cells. Autophagy-defects or deficiency may result in several ailments including neurodegenerative disorders, cardiac diseases, diabetes [4, 5], reproductive abnormalities, and even infertility [6].

The research on autophagy progressed with an outstanding pace, and to date, an ample number of autophagy-related (Atg) genes (more than 30) have been identified. There are four key events involved in autophagosome formation: (1) initiation: translocation of uncoordinated 51-like kinase 1 (ULK1) complex upon the mammalian target of rapamycin (mTOR) results in class III phosphatidylinositol 3-kinase (PI3K) activation that ultimately helps in phosphatidylinositol 3-phosphate (PI3P) formation and PI3P-binding proteins recruitment at the site of autophagosome formation. (2) Vesicle nucleation: Atg9L1 (a transmembrane protein) is involved in the nucleation event of the autophagosomal membrane. (3) Autophagosome formation: this event involved the conjugation of C-terminus of microtubule-associated protein 1A/1B-light chain 3 (LC3) with phosphatidylethanolamine (PE) for anchoring of the protein in the membrane and is mediated by a ubiquitin-like system (Atg7, Atg3, and the Atg12–Atg5–Atg16L1 complex). (4) In the final step, the isolated membrane is elongated and degraded to help the fusion of this autophagosome with lysosome [3, 7, 8].

## 2. Selective Autophagy

Autophagy triggered by food restriction was formerly considered to be a nonselective process, but a growing body of data suggests that it is, since autophagy selectively targets a variety of cargos, including organelles and protein aggregates (Figure 1). “Autophagy cargo receptors” are the main players of selective autophagy as they bind with the targeted materials having degradation signals (ubiquitin) via ubiquitin-binding domain (UBD). In mammals, the presence of an LC3 interacting region (LIR) motif equips these receptors with a unique property to interact with LC3 (Atg8) present on newly formed autophagosomes. As a result, by serving as molecular bridges, these autophagy cargo receptors designated the targeted compounds, tagged with a ubiquitin tag, for destruction by autophagy or the ubiquitin proteasome system (UPS). These receptors, such as p62/ SQSTM1, NBR1, and histone deacetylase 6 (HDAC6), support the removal and destruction of protein aggregates via UBD and LIR2 in aggrephagy (a form of selective autophagy) [3, 7–10]. Despite the lack of orthologues of UBD-containing adaptor proteins (p62) in yeast, mass spectrometric studies recently assisted in the identification of Cue5, which is likely a possible autophagy cargo receptor [11]. There is a coupling of ubiquitin conjugation to ER degradation (CUE) domain in yeast Cue5 (similar to UBDs in mammals) that can interact with Atg8 and binds to ubiquitylated targets. Therefore, Cue5 helps in the clearance and removal of ubiquitylated cargo via selective autophagy by acting as an ubiquitin–Atg8 adaptor protein (analogous to p62 in mammals). Yeast Cue5 and TOLLIP (mammalian orthologue) both aim to remove aggregation-prone proteins (i.e., huntingtin), unable to degrade via UPS, by autophagy (K. [11]). Besides the removal of protein aggregates, selective autophagy also helps in the clearance of damaged organelles (Figure 1). For instance, selective autophagic degradation of worn out and superfluous mitochondria is specifically named as mitophagy [12, 13]. Similarly, the degradation and removal of peroxisomes are known as pexophagy [14, 15]. In mammals, LC3-positive phagophores are recruited, by p62 and NBR1 (autophagy cargo receptors), to the surface of targeted monoubiquitylated peroxisomes [16, 17]. Besides mitophagy and pexophagy, selective autophagy can be categorized into various types depending upon the specific cargos to be targeted, i.e., aggrephagy, xenophagy, glycophagy, reticulophagy, ribophagy, nucleophagy, zymophagy, and lipophagy. Unlike mitochondria and peroxisomes, the detailed insight of molecular mechanism and the role of selective autophagy in the clearance of other organelles need to be elucidated. Intriguingly, ER degradation in yeast seems to be accomplished via microautophagy but in fact, no microautophagy machinery is involved in this process [18]. Moreover, large-sized macromolecules like iron complexes and lipids can also be degraded by selective autophagy. Similarly, several intermediary macromolecular species, i.e., inflammasome, midbody, and midbody ring, are also targeted by selective autophagy [19–22]. However, the only lipophagy will be discussed in the rest of this review.

## 3. Lipophagy: A General Overview

There is a battery of powerful hydrolytic enzymes in lysosomes including glycases, nucleases, proteases, and lipases that are responsible for a rapid turnover of unnecessary cell components [23]. Molecular basis of how cellular fat stores are regulated in lipid droplets (LDs) remains a hot topic in the past few years [24]. Cells tend to store the surplus energy in the form of fats, during nutrient-rich status, within special cytoplasmic structures called LDs that act as a vast depot of a variety of molecules such as esters, cholesteryl esters, neutral lipids, and triglycerides [25]. These LDs are encircled by a variety of coat proteins (generally perilipins family proteins (PLINs)) and a single layer of phospholipids [26]. These fat depots are often regarded as distinct organelles because of their ability to neutralize the effects of infectious entities [27] and misfolded proteins [28] and also because of their dynamic interactions with other cytoplasmic organelles [29]. LDs are manufactured at ER and generally range in size from 0.1 to 10 *μ*m in most of the cell types [25, 30]. However, LDs found in adipocytes (fat storage cells) may attain a much larger size than average and grow up to 100 *μ*m in size [31].

**Figure 2:**
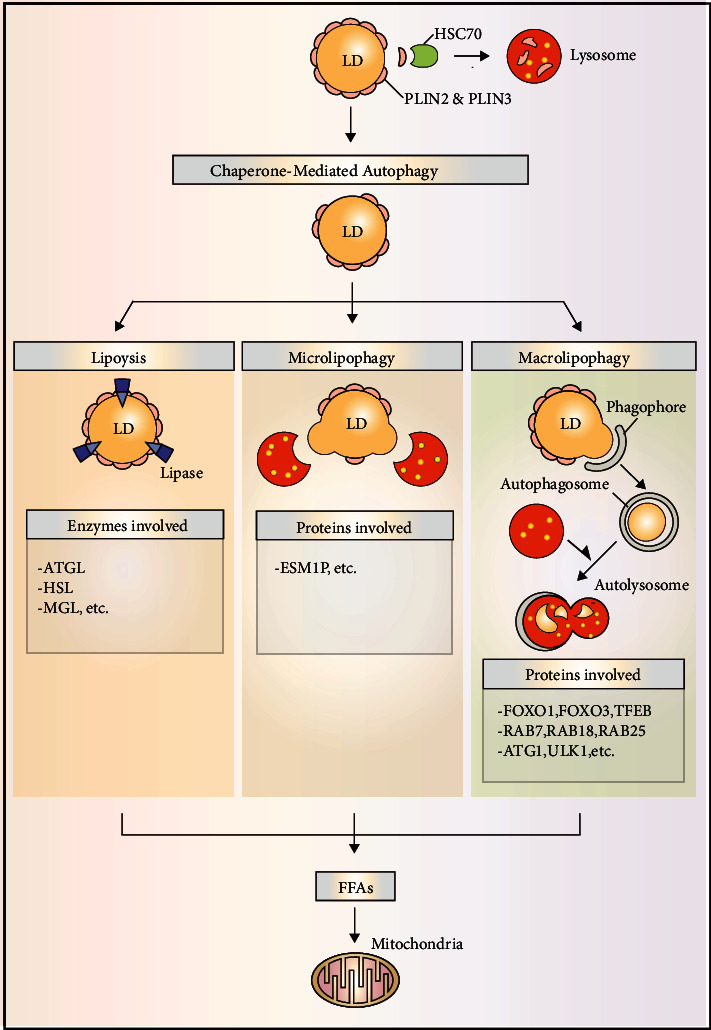
Major pathways in LD degradation: CMA facilitates LD degradation by promoting PLIN protein degradation allowing access for lipases. Moreover, both microlipophagy and macrolipophagy also degrade LDs to generate free FAs that are utilized in mitochondria for their complete oxidation.

Starvation stimulates lipids to break down in a process named as lipolysis (Figure 2) by protein kinase A activation that results in phosphorylation of PLIN1 (LD coat protein) [32]; this phosphorylation helps in degradation of PLIN1 by proteasomal activity that recruits cytosolic lipases to LDs [32]. The autophagolysosomal system was postulated to be implicated in the destruction of LDs during starvation because nutritional shortage causes autophagy activation, which supplies nutrients by breaking down worn out and unneeded cytoplasmic material via lysosomal degradation. Hepatocytes were either chemically treated with an autophagy inhibitor (3-methyladenine) or autophagy was inhibited using small hairpin (sh) RNA against Atg5 to test this hypothesis. Following that, in both situations of autophagy failure, a substantial rise in LD content was found [22]. Moreover, an increased level of triglyceride was also observed in autophagy-deficient mouse embryonic fibroblasts [22]. Further studies employing mitochondrial *β*-oxidation and rates of triglyceride breakdown revealed this increase in lipid contents was a result of decreased lipid turnover in autophagy-deficient cells instead of an increased triglyceride synthesis. In addition to it, sequestration of LDs by LC3 (autophagy marker)-labeled autophagosomal membrane was confirmed using immunogold electron microscopy. Subsequently, in the starved liver, the presence of LC3, PLIN1, and PLIN2 was also confirmed in LDs and lysosomes [22]. Furthermore, the colocalization of LDs and lysosomes indicated the significance of the lysosomal degradation system in LD catabolism [22]. As autophagosomes were found to engulf LDs and subsequently fused with lysosomes, lipophagy is regarded as selective macroautophagy (macrolipophagy) involved in LD breakdown (Figure 2). In line with these findings, selective Atg7-knock out in the liver resulted in increased LD contents *in vivo* and was comparable to nonalcoholic fatty liver disease (humans) histologically [22]. Interestingly, the acute supply of lipids (oleic acid) was found to trigger lipophagy in hepatocytes [22]. Lipophagy (autophagy) activation is perhaps an ubiquitous response against a sharp lipid influx as observed in various cell types. For instance, similar response to eliminate the excessive lipid contents was also observed in cultured hypothalamic neurons treated with oleic acid [33]. In contrast to the acute supply of lipids, chronic lipid supply showed an opposite trend, and suppression of lipophagy was observed in mice obesity models (both genetic and diet-fed models) and led to ER stress and hepatic inflammation upon prolonged high-fat-diet feeding [22]. In line with it, ATG7-overexpression successfully reversed hepatic LD accumulation and prevented steatosis in obese mice [34]. The results of all these studies are suggestive of an important role of lipophagy (autophagy) in LD turnover. However, an outstanding question that what causes the autophagic flux to decrease under over nutrition conditions remains to be elucidated. Multiple mechanisms are speculated to be involved in decreasing the autophagy in obesity. Previously, *in vivo* assay showed a 70% decrease in the fusion of autophagosome with lysosome in a high-fat diet mice model [35]. Similarly, a significant decrease in autophagolysosomal fusion was also noted in alcohol-induced hepatotoxicity [36]. Besides lysosomal pH and ATP availability, few dietary factors also affect autophagolysosomal fusion activity by altering the lipid composition of the lysosomal membrane [35, 37]. The first report describing autophagy contribution in hepatic LD turnover threw into gear a new chapter of research, and since that time, a plethora of studies have now identified lipophagy as the main player involved in LD breakdown in a variety of cells, i.e., adipose-resident macrophages [38], prostate cancer cells [39], orexigenic hypothalamic neurons [33], lymphocytes [40], cultured adipocytes [41], macrophage foam cells [41–43], primary striatal neurons, glial cells [44], and gastrointestinal epithelial cells [45]; in nonmammals, i.e., yeast [46, 47], fungus [48], and worms [49, 50]; in plants including rice [51]; and also in phyllosphere microorganisms [52]. Therefore, these findings are enough to support the use of term macrolipophagy/lipophagy as an ubiquitous pathway responsible for LD mobilization [22].

Lipophagy is a highly regulated process; recently, autophagy was found to be regulated by SNARE (soluble N-ethylmale-imide-sensitive factor-attachment protein receptors) proteins that do so by facilitating the recruitment of ATG proteins to autophagosomal formation sites [53, 54]. SNARE protein syntaxin-17 has also been reported to regulate autophagosomal fusion with lysosomes; however, whether this protein is also involved in decrease of lipophagy/autophagolysosomal fusion in obesity is still not known [53]. Moreover, membrane GTPases have been associated with LD degradation as LDs were found to be significantly accumulated in autolysosomes upon small GTPase dynamin-2 inhibition [55]. Recently, Bif-1 (a membrane curvature protein) has been identified as a novel protein regulating lipophagy in adipocytes (Y. [56]). Lipophagy induced an enhanced Bif-1-dependent PLIN1 breakdown, and its deficiency ultimately reduced triacylglycerol (TAG) hydrolysis, suggesting the significance of Bif-1 in lipophagy-dependent PLIN1 degradation and subsequently LD catabolism (Y. [56]). Interestingly, membrane curvature was shown to vary with the size of the LDs; however, whether Bif-1 regulates lipophagy via altering the size of the LDs is unknown at this time. In fact, the processes that activate lipophagy may be context-dependent and differ depending on the cell type. These lipophagic processes are adapted by cells to cope with severe conditions such as starvation and lipotoxicity. Despite these differences in processes, it is assumed that the basic machinery for lipophagic breakdown is conserved [57].

## 4. Mechanisms of Lipophagy Induction and Its Molecular Machinery

### 4.1. PLINs and Lipases in Lipophagy Induction

Despite variations in the size, composition, and location of LDs in the cell, the best feature that better defines their dynamic character is their proteome. On the surface of the LDs are a number of proteins that control their signalling and metabolization. One of these proteins' characteristics is their participation in the control of LD-related processes, particularly lipophagy. The perilipin family of proteins is one of the best-known protein families that affect LD activities and perform many tasks in LD biology [58]. PLIN family comprised of five members (PLIN 1-5) that share the varying degree of homology and differs from each other in their activities and tissue expression profiles. PLINs are capable of modulating the access of lipases to LD surface, hence regulating the LD metabolism [58]. It has been recently reported that PLINs are the major molecules that may link chaperone-mediated autophagy (CMA) to LD catabolism [59]. All of these investigations have described the accumulation of LDs upon lysosome-associated membrane protein 2A (LAMP2A) degradation which is an important protein involved in CMA. Moreover, to promote CMA-mediated breakdown in lysosomes, the binding of the heat shock cognate protein of 70 kDa (Hsc70) to CMA recognition motif (KFERQ) within PLIN2 and PLIN3 was observed (Figure 2). Reduction of lipophagy and lipolysis mediated by lipases was found to be decreased upon CMA ablation and is suggestive of the significance of PLIN2 and PLIN3 degradation in promoting LD catabolism and in allowing access of adipose triglyceride lipase (ATGL) and other autophagic proteins to LD surface. Further investigation helped in the identification of a vital protein named AMP-activated protein kinase that is involved in CMA-mediated degradation of phosphorylated PLIN2 [60]. As a result of these findings, it can be inferred that PLIN degradation through CMA is an upstream process necessary for lipophagy start. The importance of ATGL in the catabolism of TAG during the degradation of PLINs may also be deduced from the aforementioned explanation. More in-depth research later revealed a strong connection between autophagy (lipophagy) and ATGL. For instance, a direct interaction between LC3 and ATGL has been demonstrated on the LD surface [61] along with LC3 interactions with HSL. This interaction between LC3 and ATGL occurs at LIR residues 145-150 (STFIPV). LIR depletion caused a deficiency in ATGL localization and translocation to the LD surface during hunger. These findings point to LC3 playing a crucial role in TAG hydrolysis by transporting ATGL to the LD surface. However, it is uncertain why ATGL requires LC3 for its localization to the LD surface.

According to the studies carried out to date, it can be perceived that both ATGL and lipophagy contribute directly to LD catabolism (Figure 2). Sathyanarayan and colleagues have recently described if there any linearity exists between ATGL and autophagy/lipophagy [62]. ATGL was found to promote the interactions of LC3 and lysosomes with LDs and positively regulates autophagic flux in the liver suggesting enhanced lipophagy. Intriguingly, the effects of ATGL overexpression on LD breakdown were diminished as a result of lysosomal acid lipase (LAL) degradation or genetic/chemical inhibition of autophagy. ATGL was previously shown to promote SIRT1 (an important autophagy player) activity [63, 64], and SIRT1 was also found to mediate the effects of ATGL in endorsing autophagy (lipophagy). Furthermore, we recently reported on Sirt1's function in testosterone production via autophagy control in the LC3-dependent pathway. Sirt1 promotes autophagosome production by deacetylating LC3 in the nucleus of leydig cells. NHERF2 is degraded by autophagosomes, which increases the expression of SR-BI. By increasing cholesterol absorption, SR-BI speeds up testosterone biosynthesis [65].

Subsequently, autophagy (lipophagy) was found to be increased by PNPLA5 and PNPLA8 (two members of ATGL (PNPLA2) containing family) in the variety of cell types [66, 67]. It has been described that diacylglycerol produced by PNPLA5 is involved in the synthesis of autophagosomal membranes, hence regulating protein trafficking by influencing membrane curvatures. Concluding the above results, it can be interpreted that lipases including ATGL regulate autophagy (lipophagy) and the resultant lipophagy mediates bulk degradation of LDs. It is noteworthy that impaired lipophagy and normal macroautophagy observed in macrophages lacking ATGL are indicative of the existence of cell-type-specific function [68]. Although lipolysis is tightly regulated via multiple signaling pathways (cAMP/PKA, AMPK, etc.) and a vast variety of proteins influence it via direct interaction with ATGL or other LD proteins, much remains to investigate about how lipophagy is regulated via ATGL-mediated signaling.

### 4.2. Rab GTPases in Lipophagy Induction

Many Rab proteins have been known to play critical roles in LD metabolism and LD biology since the time of their discovery [69–72]. Around 70 members of Rab family GTPases are thought to regulate endosomal membrane trafficking via conferring a special “identity” to trafficking items [73]. Rabs serve as molecular switches, cycling between active (GTP-bound) and inactive (GDP-bound) states. By controlling the connections between membrane fusion complexes and cytoskeletal components, this interconversion is critical for enabling the complicated intracellular vesicular traffic (motor proteins). The discovery of over 30 Rab GTPases on the surface of LDs has sparked a fresh wave of research into their role in LD catabolism [74]. An impaired lipophagy, characterized by a pronounced decrease in LD turnover, was observed upon the ablation of several members of the GTPases family. Rab7 is an important player in autophagosomal maturation [75, 76] and intracellular trafficking (a marker of the endocytic pathway) [77], one of those Rabs that concentrate on LD surface. Rab7 facilitates the interactions between components of HOPS tethering complex and numerous SNARE proteins hence promote the fusion of endocytic and autophagic membranes [78, 79]. Moreover, Rab7, along with several downstream effector components including FYVE, FYCO1, and RILP, mediates organelle transport (both plus- and minus-end directed) in tight cooperation with kinesin and dynein-dynactin motors [80–82]. Therefore, Rab7 is thought to play a prime role in mediating interactions among many cell components and actively contributes to promoting versatile biological activities such as autophagy (lipophagy). Interestingly, Rab7 has also been found to play an important role in mitophagy where it facilitates the encapsulation of mitochondria within autophagosomes with the help of TBC1D15 and TBC1D17 (two significant GTPase-activating proteins) [83]. Rab7's enhanced colocalization with LDs, lysosomes, and autophagosomal membranes led to the discovery of Rab7's important involvement in LD homeostasis for the first time [84]. *Rab7*-knockdown via siRNA or a negative form of Rab7 in hepatoma cells led to an abnormal LD accretion [85]. During starvation, Rab7 facilitates the priming of LDs by increasing GTP affinity than GDP and recruiting the degradative machinery to LD surface by directly activating on LD surface [86]. The findings of this study point to Rab7's importance in regulating hepatic lipophagy by serving as an LD-localized node. Rab7 has recently been linked to ethanol-induced hepatic steatosis as well [87]. Rats on an alcohol-rich diet were found to be resistant to starvation-induced lipophagy and developed hepatic steatosis as a result. Rab7 levels were substantially lower in hepatocytes treated with alcohol compared to controls, indicating an autophagic failure in LD detection. Furthermore, more research is needed to determine the specific mechanism behind the inhibition of lipophagy in ethanol-exposed hepatocytes. Rab10, which is found on the surface of LDs, is another member of the Rab family that may play a role in lipophagy. This Rab member regulates Golgi trafficking during epithelial polarization [88] and insulin-stimulated GLUT4 vesicular trafficking [89]. Furthermore, in a recent report, Rab10 has been found to play a key role in ER morphogenesis by regulating tubular extension and fusion [90]. Rab10 depletion or genetic ablation also led to an increased LD accumulation in hepatoma similar to Rab7 [91]. In starved cells, there was an interesting translocation and colocalization of Rab10 (activated) with autophagic membrane markers such as LC3 and Atg16 on the LD surface. Rab10 may enhance LD envelopment by increasing phagophore via EH domain-binding protein 1 and 2 (membrane-deforming ATPase) functioning in a complex downstream of Rab7 during lipophagic development; it was hypothesized [91]. Although other members of the Rab family have also been associated with lipolysis and LD metabolism, however, their putative roles in LD selective autophagy are largely unknown. Colocalization of Rab32 with LD and autophagic membrane markers have also been reported in the fat body of *Drosophila* [92]. Furthermore, an increased ATGL level and the decrease in number and size of LDs was found upon *Rab32*-knockdown [93]; however, how ATGL expression was increased remains unidentified and need more in-depth studies. Another member of GTPases known to contribute in LD homeostasis is Rab18. Rab18 was found tightly associated with LD surface and found to influence adiposity [94]. Upon *β*-adrenergic stimulation, most of this small GTPase (Rab18) was exclusively found to localize at LD surface [95, 96]. Rab18 binding to only a subset of LDs indicates a distinct recruitment pattern that is dependent on the metabolic state of the LDs. Interactions between the proteins COP-I and TRAPP-II aid in the relocalization of TRAPP-II on the surface of LDs and enhance lipolysis by activating Rab18 [97]. TRAPP complexes have been described to participate in autophagy [98]; hence, there is a need to carry out further investigations to explore any possible role of Rab18-TRAPP interactions in lipophagy. In addition to these studies, Rab25 have been recently identified in hepatic stellate cells (HSCs) to contribute in autophagy of LDs enriched in retinyl ester (Z. [99]). The increase in Rab25 expression was assumed to be caused by the release of reactive oxygen species (ROS) as a result of HSC activation. Rab25-ablation by siRNA led in LD turnover being reduced, which inhibited HSC activation. These findings point to a new role for Rab25 in lipophagy; however, it is unclear if this GTPase works just on the surface of LDs or impacts other autophagic arms.

### 4.3. LD-Surface Receptors in Lipophagy

Identifying the receptors involved in cargo-specific selective autophagy has been of significant interest in revealing the link between a particular organelle and autophagic machinery [9]. With the exception of LDs, these unique autophagic receptors have been identified in nearly all organelles. The majority of these receptors were discovered to interact with LC3B via LIR, as previously reported [100]. For example, the discovery of NDP52 and optineurin (two of the most essential mitochondrial cargo receptors) provided fresh and interesting insights into mitophagy [101, 102]. NDP52 may participate in other types of autophagy (i.e., xenophagy) [103, 104] while there are several other general candidate receptors such as p62/ SQSTM1, NBR1, and Huntingtin that play important roles in targeted turnover of several other organelles [16, 105, 106]. Furthermore, significant accumulation of LDs as a result of a mutation in Huntingtin is suggestive of a critical role of this receptor protein in LD metabolism [106, 107]. Because LC3-binding to ATGL promotes LD degradation, no additional LD-resident proteins that unequivocally mediate lipophagy via an LC3-binding mechanism have been discovered yet. These findings point to other proteins (such as Rab7 and others) recruiting autophagic machinery via an as-yet-unidentified mechanism. Protein changes such as polyubiquitination are an alternate method for promoting lipophagy. For example, ancient ubiquitous protein 1 (AUP1) can be marked for destruction by interacting with Ube2g2 via its G2 binding domain (E2 ubiquitin conjugase G2) [108–110]. To understand the details of how this signaling is initiated and how it helps in regulating LD turnover via autophagy required more rigorous investigations on this front in the near future.

## 5. Lipophagy Regulation

### 5.1. Transcriptional Regulation

Over the last few decades, considerable progress has been made in understanding autophagy and, to a lesser degree, lipophagy regulation at the transcriptional level. Microphthalmia-associated/TFE subfamily of basic/helix-loop-helix/leucine zipper transcription factors, including HLH-30 (*C. elegans*) and TFEB and TFE3 (mammals), are the most researched autophagy/lipophagy regulators among all of these transcriptional regulators. TFEB has been discovered to enhance lipophagy as well as contribute to fat metabolism by activating PPAR-coactivator 1 (PGC-1) and PPAR-targeted genes that govern fat catabolism [111]. Lipophagy and lysosomal-associated lipolysis were observed as a result of coordinated TFEB homolog HLH-30 activation that does so by enhancing the expression of many lysosomal lipases in *C. elegans* [50]. Moreover, starvation-induced LAL expression was found to be TFEB dependent in mice. In addition to it, TFE3 has also been found to regulate hepatic lipophagy. It is of great interest that steatosis was observed upon hepatic ablation of TFE3 while hepatic overexpression of TFE3 resulted in a decrease of steatosis via lipophagy induction [112]. TFE3 overexpression in adipocytes, on the other hand, was shown to increase obesity [113], which may be explained based on current research showing the important role of autophagy in adipocyte development ([114, 115]; C. [116]). These studies are suggestive of differential regulation of lipid metabolism by TFE3 in adipocytes and liver. Lipophagy induction and LAL expression were noted in adipose tissue as a result of starvation-induced activation of FOXO1 [41]. Furthermore, hepatic steatosis and hypertriglyceridemia were observed in liver-specific FOXO 1/3/4 knockout mice while overexpression of ATG14 resulted in a reversal of these anomalies [117]. In a similar research, cAMP response element-binding protein (CREB) was found to enhance lipophagy under deprivation by activating the TFEB, but food supplementation reversed these circumstances via farsenoid X receptor activation (FXR) [118]. Moreover, PPAR-*α* activation under nutrient depletion was found to inhibit FXR-mediated lipophagy suppression (J. M. [123]), and the results of this study speak about a regulatory interaction between FXR and PPAR-*α*. Based on these findings, it can be concluded that starvation-induced activation of certain transcription factors and coactivators such as TFEB, TFE3, PPAR-*α*, PGC-1*α*, FOXO1, and CREB is involved in promoting while nutrient supplementation-induced transcription factors, such as FXR, are involved in suppressing lipophagy. Recently, it was shown that the sterol response element-binding protein-2 (SREBP-2) is also involved in autophagy/lipophagy control. SREBP-2 knockdown activates autophagy and increases LD turnover by increasing autophagosome formation in the presence of sterol deprivation [119]. In addition to it, SREBP-2 was found to prevent liver steatosis by promoting lipophagy via enhancing the expression of PNPLA8 [67]. Therefore, based on these findings, it can be concluded that SREBP-2 compensates cholesterol deficiency not only by promoting lipophagy but also by directly increasing the biosynthesis of cholesterol by promoting the expression of the relevant genes. Furthermore, these studies throw the light on the significance of lipid signaling in lipophagy regulation.

### 5.2. Nutritional and Hormonal Regulation

Various mechanisms that respond to the nutrient status of cell also regulate lipophagy, such as mTOR (H. [120]), AMPK (Y. [121]), farnesoid X receptor (FXR), and peroxisome proliferator-activated receptor-*α* (PPAR*α*) (J. M. [122]). mTOR is engaged in a variety of activities, including the control of several downstream metabolic processes. It is also known as the major signalling hub since it reacts to a variety of hormones, including insulin, as well as nutrients like glucose and amino acids. It is a strong autophagy inhibitor that is activated in response to nutrition. When rapamycin was used to block mTOR via autophagy activation, enhanced lipid oxidation and catabolism were found (mTOR inhibitor) [123]. In addition to it, LD turnover was observed to be increased in hypothalamic neurons upon autophagy induction via serum deprivation that inactivates mTOR by decreasing its phosphorylation [33]. Moreover, the interplay between the autophagic process and lipolysis via mTOR inhibition has been described in an outstanding study carried out in worms [49]. Interestingly, rapamycin was found to increase the lifespan of *C. elegans* via enhancing the activity of lysosomal lipase lipl-4. Therefore, it can be concluded that mTOR similarly regulates lipophagy and autophagy; however, how it suppresses lipophagy that remained largely unknown?

It is of great interest that hormones that are usually involved in catabolism also contribute to promoting lipophagy. For instance, *β*-adrenergic signaling was found to enhance the lipophagic process in adipose tissues similar to hepatocytes [85] in a Rab-7-dependent manner [84]. Thyroidine (T3) is another important hormone whose role in hepatic mitochondrial *β*-oxidation and LD catabolism has been well studied. Moreover, the presence of functional thyroid receptors was found to be necessary for LD turnover via autophagy (Rohit Anthony [124]). However, how lipophagy is regulated by T3 in extrahepatic tissues requires further more detailed investigations?

Furthermore, lipophagy activation by oleic acid is suggestive of lipophagy regulation by lipids themselves [22]. This activation of lipophagy was thought to be a cellular response to eliminate the influx of excess lipids. Intriguingly, Kaushik et al. [33] reported similar findings in hypothalamic neurons as a result of oleic acid treatment, suggesting this response as a ubiquitous effect observed in numerous cell types [33]. On contrary to acute supplementation, chronic fat supplementation resulted in steatosis characterized by low LC3 level in LDs, and a reversal of these circumstances was observed upon hepatic ATG7 restoration (L. [34]). Furthermore, chronic fat supplementation was found to decrease lipophagy by reducing LAMP-2A that subsequently causes a reduction of PLIN2 degradation via CMA [125].

### 5.3. Lipophagy Regulation via Small Molecules

A vast variety of natural compounds modulate lipophagy. A polyphenolic compound, abundantly found in green tea, named epigallocatechin-3-gallate (also called EGCG) has been observed to influence hepatic autophagy/lipophagy [126, 127]. Similarly, caffeine was found to protect fatty liver disease by promoting LD catabolism via lipophagic induction [128]. Furthermore, a dietary polyphenol contained in the peel of the bergamot citrus fruit (bergamot) was shown to regulate hepatic steatosis. In the livers of rats given 50 mg/kg bergamot for three months, there was a better LD output as well as increased interactions between autophagic machinery and LDs [129]. Furthermore, other elegant studies reported the protective role of red wine bioactive resveratrol in decreasing the extent of hepatic steatosis via autophagy/lipophagy mediation (Y. [130]). In contrast to above-described molecules that promote autophagy/lipophagy, tetrandrine, a bisbenzylisoquinoline alkaloid, resulted in lipid accumulation in the liver cell line by impairing the normal autophagic process. Although a range of natural compounds has been reported to modulate autophagy, however, whether these compounds also regulate lipophagy is unknown yet [131].

## 6. Lipophagy in Disease States

### 6.1. Lipophagy and Pathophysiology of the Liver

Lipophagy is a key regulator of LD metabolism in hepatic homeostasis; as a result, any disruption in its normal function can cause to excessive lipid buildup or steatosis, which can manifest in both alcoholic and nonalcoholic fatty liver disease (NAFLD) [132]. Furthermore, lipophagy also modulates inflammation and apoptosis, major characteristics of these ailments [133]. In the first-ever study of lipophagy, hepatic TG and cholesterol level was found to be increased in hepatocytes of mice with defective autophagy [18]. Aging, obesity, inflammation, and nonalcoholic steatohepatitis all show similar deficits defined by autophagy abnormalities (NASH). Autophagy suppression resulted in a rise in steatosis in mice given a methionine-choline diet, whereas autophagy activation resulted in a significant decrease in steatosis [134]. Autophagy activation by resveratrol was also observed to attenuate the steatosis induced by this diet, suggesting a possible therapeutic potential of this compound to treat NASH [135]. Alterations in lipids other than hydrolyze triglycerides (TGs) may influence the autophagy in fatty liver disease. For instance, an increased level of bioactive sphingolipid ceramide was found in the liver of Atg7-ko mice [136]. Moreover, an increase in *de novo* sphingolipid synthesis resulted in an upregulated autophagy activity suggesting that LD catabolism via autophagy activation consume excessive LDs for liver lipid homeostasis. Similarly, ceramide was found to be upregulated in several other ailments including diabetes [137] and obesity [138], both of which are associated with steatosis development [139]; therefore, it can be speculated that autophagy defect may be a leading cause of pathologies related to altered sphingolipid contents. In agreement with these investigations, knockdown of Atg14 ([117]) or Tfeb-ko [111] resulted in the enhancement of hepatic TG level. Moreover, autophagy enhancement in obese mice via Atg7/Atg14 overexpression alleviated the steatosis extent [34, 91]. Although there is a huge number of studies in favor of alleviation of hepatic LD accumulation by lipophagy, however, some mouse models showed no or less steatosis upon autophagic defect [140, 141]. This incongruity may be a result of differences in experimental setups and animal models. It was suggested that lipophagy perhaps replenish LDs with fatty acids (FAs) during starvation *in vitro*, while lipids are removed from LDs by lipolysis [142]. However, this study was conducted on fibroblasts with low lipid content, which were not grown in a serum-free medium but rather in a saline solution for a longer period of time. As a result, study's findings are unworthy of consideration and remain unclear. As a result, in this circumstance, more comprehensive in vivo studies are needed to determine whether lipophagy reduces hepatic damage and steatosis in NAFLD patients. In acute ethanol-induced in vitro and in vivo models of liver steatosis and damage, LD degradation was revealed to be mediated via autophagy [143]. ROS intermediates, produced as a result of acute alcohol consumption, were found to induce lipophagy [143]. Similar observations were found in mice fed on ethanol diet or upon overexpression of CYP2E1 (prooxidant enzyme) ([144]). Furthermore, a lower level of the main antioxidant glutathione in the liver of mice given an alcoholic diet, which was further reduced when autophagy was inhibited, clearly implies that autophagy plays a protective role against chronic alcohol-induced damage. On the basis of the aforementioned, it can be inferred that LD degradation via autophagy protects against ethanol-induced liver damage and oxidative stress in both acute and chronic forms. This protective role may be a result of excess lipid removal that acts as a substrate for oxidative stress or via ATP supplementation generated as a result of free fatty acids (FFAs) catabolism via lipophagy, that is, the possible mechanism of resistance adopted by hepatic cells against oxidative stress [145]. ATGL and hormone-sensitive lipase (HSL) are the major factors responsible for the hydrolysis of triglycerides in adipocytes. Though ATGL shows highest expression in fat tissues, a lower level of activity is also found in several nonadipose tissues. ATGL deficiency led to an enhanced LD accumulation in liver and heart besides adipose tissues in mice. However, ATGL is involved in varying degrees of phospholipid hydrolysis while no hydrolysis of cholesteryl esters was observed under various experimental conditions. Furthermore, HSL is expressed normally at an extremely low level in liver, and there are no studies so far describing an association between HSL and lipolysis in the liver. On the other hand, a reversal of hepatic steatosis was observed upon HSL overexpression *ex vivo* [146]. “Two-hit theory” is the age-old and reliable model for the study of development and progression of NASH ([147]; [148]). The first strike is defined by the onset of fatty liver disease, followed by the second hit, which is characterised by the accumulation of reactive oxygen species (ROS), liver inflammation, and mitochondrial dysfunction, all of which lead to NASH. However, the exact mechanism by which autophagy mediates the two-hit scenario for NASH development is yet unknown. Lipophagy was initially discovered to prevent excessive fat accumulation during the first hit [22]. Secondly, autophagy helps in the elimination of ROS-producing mitochondria and maintaining ATP levels preventing the second hit. TNF-*α* is an inflammatory molecule that mediates hepatic injury, and it has been reported that Atg-7 deficiency led to hepatic injury upon administration of common hepatic toxicants including galactosamine combined with LPS/galactosamine with TNF-*α* to mice [149]. Lipophagy plays an important role in protecting hepatocytes against lipotoxicity. SIRT3, a NAD^+^-dependent deacetylase, promotes macroautophagy through AMPK-dependent pathway, downregulates SCD1 to inhibit lipogenesis, and activates LCAD to promote *β*-oxidation of fatty acids and in this way mitigate lipotoxicity ([150]). Impaired lipophagy is associated with advancement in NAFLD [151]. Recently, another study conducted by [152] have reported that dietary intake and exercise help in controlling NAFLD by enhancing lipophagy. These therapies enhance lipophagy in liver by downregulating Akt/mTOR/ULK1 pathways and upregulating AMPK/ULK1 pathways ([152]). Moreover, lipophagy not only prevents hepatic lipotoxicity but also helps in VLDL production by providing free fatty acids. In FFA-deficient conditions, nearly 70% of secreted VLDL comes from the TG degradation in hepatocytes; however, mechanism of TG breakdown to make VLDL remains unclear [153]. Moreover, autophagy inhibition resulted in reduced fatty acid oxidation and VLDL production, and autophagy activation led to an enhanced VLDL production [154, 155]. In human subjects, autophagy examination has always remained a major restraint to truly find the extent of autophagy defect in human NAFLD. In addition to it, the samples need to be pretreated with lysosomal inhibitors to measure autophagy flux and activity [156, 157]. Despite these constraints, correlative studies have provided evidence of the contribution of autophagy in the prevention of NAFLD development in humans. For instance, autophagy cargo p62/SQSTM1 was found to be accumulated in liver sections from expired subjects and in biopsy samples obtained from subjects with severe steatosis [158, 159]. As a result, an inverse relationship between autophagy and liver total lipid concentration may be hypothesised. Furthermore, autophagy inhibition by pharmaceutical drugs used to treat steatosis reinforces the link between steatosis and lipophagic activity. For example, thymidine analogues (stavudine and zidovudine), which are frequently used as antiretroviral treatment, have been shown to reduce autophagy and may be responsible for the elevated LD content in these patients' livers [160]. On the contrary, steatosis development was prevented by anticonvulsant carbamazepine which is capable of activating autophagy. Recently, obesity and related lipotoxic states have been found to inhibit autophagy perhaps due to an increased cytosolic calcium level [161]. Moreover, this calcium led to an impairment of autophagosome-lysosome fusion [161]. To further dig out this finding, obese mice were administered calcium channel blocker verapamil that resulted in autophagy activation and decreased the LD accumulation and in turn improved insulin sensitivity [161]. Collectively, these investigations from humans and mice models speak about a major function of lipophagy in LD homeostasis in liver and formulate the basis to fully understand the molecular mechanisms responsible for lipid metabolism mediated by lipophagy (autophagy) [162].

### 6.2. Lipophagy in Stellate Cell Activation and Fibrosis

Lipophagy transforms stellate cells into matrix-producing myofibroblasts, resulting in fibrosis as a pathologic condition. In their dormant condition, these stellate cells tend to accumulate more lipids, mostly in the form of vitamin A. During the activation of stellate cells, these lipid reserves are digested. In hepatic stellate cells, fibrotic stimuli were observed to increase macroautophagy [163, 164]. Autophagy-defective stellate cells were failed to activate, and restoration of activation potential upon FFA oleate supplementation is suggestive of lipophagy-driven transdifferentiation upon substrate supplementation to obtain energy required for their activation [163]. Moreover, a decrease in fibrotic extent was observed *in vivo* in autophagy-deficient stellate cells [163]. Whether lipophagy is responsible for fibrosis in organs other than the liver is still unknown; however, the prime importance of stellate cell activation in fibrosis development and progression is suggestive of significant roles of lipophagy in this cell that may be critical in some fibrotic disorders. Taken into account the previously described role of lipophagy in liver steatosis, fibrosis, and injury, it can be suggested that autophagy may influence multiple hepatic features of NAFLD affecting a variety of cells [165].

### 6.3. Lipophagy in Metabolic Disorders

The finding that autophagy functions in adipogenesis have thrown in gear a considerable interest in investigating whether any possible association exists between autophagy and metabolic disorders, i.e., obesity. A direct correlation was found between autophagy level and fat depot size among human subjects with varying degrees and types of obesity. Intriguingly, significantly high autophagic activity was observed in omental fat tissue samples from obese subjects, and as a matter of fact, a further increase in autophagy extent was found in obese individuals with insulin resistance [166]. In addition to the functional involvement of autophagy in the differentiation of adipose tissues during development, our findings imply that autophagy may play a role in controlling the size of adipose tissues and lipid homeostasis in adulthood. The increase of autophagy prior to obesity-related morbidity suggests that autophagy activation may be used as a defence mechanism to deal with excessive cellular lipid levels. Autophagy's effects and outcomes, on the other hand, might vary depending on metabolic status. For example, mTOR attenuation has recently been reported in adipocytes from type 2 diabetes patients, and this discovery lends credence to the idea that this might be the mechanism behind autophagy increase in these cells. As a result of the accelerated production of LDs and their increased autophagic activity, excessive FFA is released from LDs, posing cellular toxicity. In these cases, autophagy blockage or autophagy decrease may be a preferable technique [167]. Lipophagy has been put forward recently as a probable defensive strategy to prevent atherosclerosis resulting from atypical lipid amassing in macrophage foam cells [168]. Furthermore, lipolytic LD mobilization in foam cells mediated by autophagy was also proposed as a potential mechanism responsible for atherogenesis. Lipid loading resulted in an increased macrophage lipophagy both *in vitro* and *in vivo*, and an abnormal cholesterol removal was observed upon autophagy defect in mice [42]. It can be speculated based on these findings that higher concentrations of these circulating lipids may impair the autophagic activity of macrophages and resulting in intracytoplasmic lipid deposits transform them into “foam cells” [35]. The subsequent lipid deposition is the seeding to favor atherosclerotic plaque development. Therefore, present therapeutic attempts are targeting macrophages to reduce the extent of lipid buildup in the form of endothelial plaques by favoring the cholesterol efflux from them. Consequently, interventions aimed at promoting autophagy and enhancing cholesterol efflux are promising therapeutic tools in the cure of atherosclerosis. Interestingly, the potential of arterial smooth muscle cells may also be utilized in the manipulation for autophagy upregulation to reduce arterial plaque formation. Furthermore, the inflammation associated with plaque formation is a consequence of the compromised autophagic activity of these cells [169].

### 6.4. Lipophagy in Aging and Longevity

**Figure 3:**
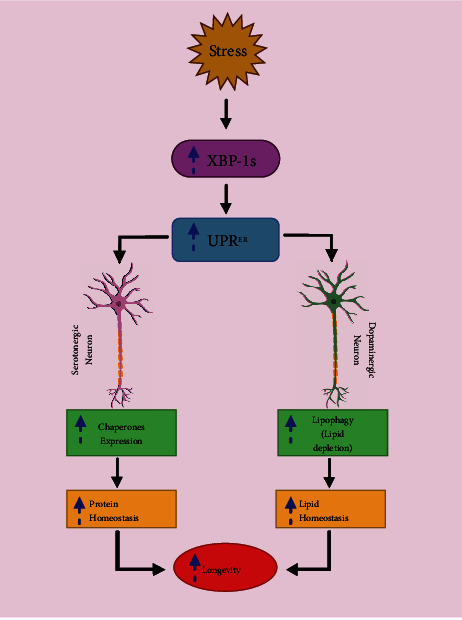
XBP-1s enhances longevity by promoting lipid and protein homeostasis via two independent pathways mediated by UPR^ER^. XBP-1 induction promotes protein homeostasis via chaperones and lipid homeostasis via restructuring ER. This restructuring enhances lipophagic depletion of LDs.

The autophagy process was found to be declined in many of the tissues and organisms as the aging process goes on [170]. This decrease in autophagy, particularly lipophagy, resulted in the buildup of LDs, and the resulting large lipid depots exacerbated the situation by further impeding the autophagic process, favouring a path for the metabolic syndrome of ageing via a potential feedback loop. Massive lipid accumulation in organs, hypercholesterolemia, and insulin resistance are all symptoms of the metabolic syndrome of ageing. Antilipolytic medicines have demonstrated the ability to ameliorate not just hypercholesterolemic conditions but also general health span in mice models of ageing, which is of considerable interest [171]. However, their beneficial effects are thought to be a result of autophagy induction in response to increasing cytoplasmic lipid contents [172, 173]. Genetic associations among autophagic process, longevity, and atherogenesis have been found recently in *C. elegans* [49]. Functional autophagy was found to activate LAL-4, and in turn, this lipase showed a potential of autophagy induction. Interestingly, the autophagic process and lipase need to work together for lifespan extension upon germline elimination. Though the particular target and how it affects autophagy remains unknown, it can be speculated that it contributes in longevity and lifespan extension by better handling of cytoplasmic lipid contents via lipophagy [174]. Recent studies have found that unfolded protein response of the endoplasmic reticulum (UPR^ER^) extend lifespan in an *xbp-1s-*dependent manner [175] which is unregulated in response to stress [176]. UPR^ER^ reverses onset of aging and extends life span by two independent pathways. UPR^ER^ regulates protein homeostasis via upregulating the expression of chaperones; on the other hand, it enhances depletion of lipid droplets by promoting lipophagy via restructuring ER morphology [177, 178]. Moreover, it has been found that dopaminergic neurons activate UPR^ER^ to drive lipid homeostasis, and serotonergic neurons drive protein homeostasis by upregulating UPR^ER^. These two events in turn enhance longevity (Figure 3)

### 6.5. Lipophagy and Viral Replication

**Figure 4:**
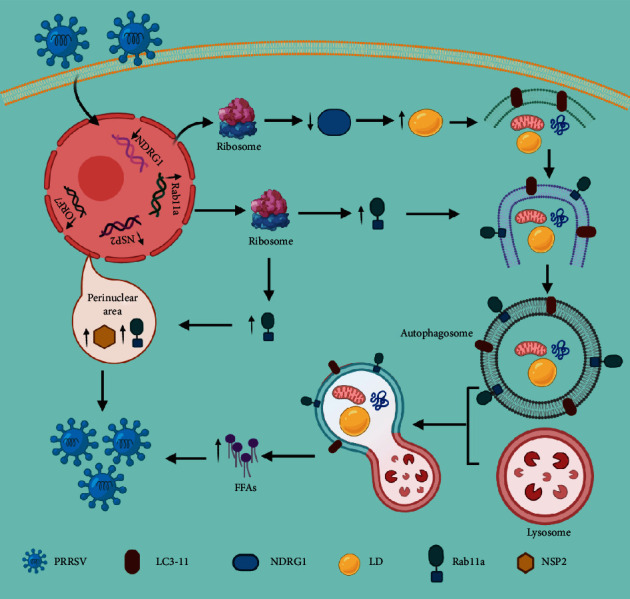
PRRSV infection promotes downregulation of NDRG1 and upregulation of Rab11a to promote replication: viral infection reduces the expression of NDRG1 and enhances expression of Rab11a, both in turn promote lipophagy by inducing autophagy and enhancing autophagosomes maturation respectively. Lipophagy promote viral replication by providing FFAs via LD degradation.

Removal of superfluous cellular elements by autophagy is suggestive of its involvement in the elimination of intracellular pathogens [172]. In contrary to popular belief, certain infectious agents may be able to manipulate this degradative route to their advantage. Hepatitis viruses are the most common cause of chronic hepatitis, and they modify autophagy in host cells by an unknown mechanism. HCV causes an increase in autophagy [179, 180] and HBV [181, 182], and this augmentation of autophagy helps in the replication of viruses instead of their elimination. An association exists between the HCV core proteins and LDs that helps in viral assembly via an unknown mechanism [183]. Similarly, autophagy augmentation has also found to enhance the replication of the dengue virus via the lipophagic mechanism. LD-associated replication, similar to HCV, has also been found in the dengue virus; however, the site of assembly remains ambiguous [184]. It has been recently described that autophagy may provide the energy required for viral replication by metabolizing the lipids [185]. In hepatoma cells, the dengue virus supported LD-coupled autophagosome development. In infected hepatoma cells, lipophagy resulted in an increase in -oxidation and a significant drop in LD volume and TG. Furthermore, inhibiting autophagy caused an increase in -oxidation levels, which halted viral propagation. When FFA was added, the replication barrier was removed, indicating that autophagic breakdown of LDs resulted in the release of FFAs that were metabolised by -oxidation to enhance viral replication. Replication of HCV has also been shown to be dependent on *β*-oxidation [185]. In hepatocytes, induction of lipophagy by these viruses is a key event in the viral life cycle and may serve as a potential novel therapeutic target for future therapies. The exact mechanism involved in viral induction is still unknown; however, NS4A has been identified as a potential candidate involved in its induction [186]. Autophagy was found to restrict the replication of dengue virus in monocytes; therefore, it can be speculated that autophagy induced by viruses may vary in other types of cells [187]. Moreover, further investigations are required to explore whether lipophagy is involved in the replication of the viruses that alter the autophagic function such as HBV (K. [188]). According to Wang et al. [189], PRRSV infection increases the expression of Rab11a at both the mRNA and protein levels. Increased Rab11a expression promotes autophagosome maturation, which leads to lipophagy. NSP2 and ORF7 expression was decreased when Rab11a expression was disrupted. Rab11a silencing resulted in an accumulation of LC3-II and p62, indicating Rab11a's function in autophagy surge. Furthermore, NSP2 and Rab11a were discovered to be colocalized, indicating that Rab11a aids viral replication by functioning as a proviral host factor (K. [189]). Recently, it was found that downregulation of NDRG1 expression promote PRRSV (Porcine reproductive and respiratory syndrome) replication by promoting lipophagy which in turn increase the level of FFAs by increasing LD degradation (Figure 4) ([190]).

## 7. Conclusion

Considering the facts described above, it can be inferred that autophagy is engaged in the metabolism of a range of substances, with cytosolic lipids lately being added to the list of targets. In the last decade, research on the function of autophagy in lipid metabolism has advanced our understanding of how autophagy uses lysosomes to maintain lipid homeostasis. The specific molecular processes of most of the major activities involved in LD trafficking and catabolism, as mentioned above, are, nevertheless, largely unknown. Despite several studies, many unsolved issues remain, including (1) how lipids are selectively directed towards lysosomes? What are their molecular routes, exactly? (2) How can lysosomes distinguish between different types of lipids? (3) How does lipophagy break down particular lipid species? (4) How is the equilibrium between various lipid catabolic pathways maintained? (5) How can the integrity and cross-talk between selective and nonselective LD breakdown through autophagy be maintained? Furthermore, because the lysosome is the primary destination of autophagy and other endocytic processes, identifying particular proteins that regulate lipophagy might have therapeutic implications. Because autophagy (lipophagy) dysfunction is linked to a range of pathological disease states, additional in-depth research to uncover the specific molecular processes is recommended.

## Data Availability

There is no additional supporting data in this article.
